# A novel CD69 binding ankyrin repeat protein (Ankyron^™^) enables detection of early activated and antigen-specific T cells in pigs

**DOI:** 10.21203/rs.3.rs-9722577/v1

**Published:** 2026-06-01

**Authors:** Leonie Bettin, Tamara Borysova, Theresa Dangl, Juan B. Odasso, Tobias Käser

**Affiliations:** University of Veterinary Medicine Vienna; University of Veterinary Medicine Vienna; University of Veterinary Medicine Vienna; University of Veterinary Medicine Vienna; University of Veterinary Medicine Vienna

**Keywords:** Pig, CD69, Ankyron, Ankyrin repeat protein, activation marker

## Abstract

T cells are essential for a wide range of protective immune responses, but significant gaps remain in our understanding of their phenotypic and functional diversity, especially in large animal models, like the pig. This is partly due to the limited availability of porcine-specific reagents and validated markers to study T-cell activation and differentiation. One marker involved in both of these processes is CD69: In mice and humans, it serves as an early activation marker and is used to define tissue-resident memory T cells. However, its use for the identification and functional assessment of T-cell subsets in pigs has been previously restricted by the lack of monoclonal antibodies. To address this limitation, we evaluated an alternative approach to antibodies: A novel ankyrin repeat protein, Ankyron^™^ EG40360, was used as a tool to detect porcine CD69. Ankyrin repeat proteins offer advantages over conventional antibodies, including their small size, improved tissue penetration, and simplified integration into flow cytometry panels due to the absence of isotype restrictions. We confirmed specific binding of Ankyron^™^ EG40360 to porcine CD69 using both flow cytometry and microscopy in porcine CD69-transfected HEK293T cells. Thereafter, we studied CD69 expression in PBMCs stimulated with PMA/ionomycin. While CD69 was basically absent in non-stimulated control cells, CD69 was highly expressed upon PMA/ionomycin stimulation as early as 4 hours post stimulation. Thereby, we could confirm CD69 as an early marker of lymphocyte activation in pigs. To further assess the expression of CD69 with the Ankyron^™^ EG40360 in a more natural setting, we studied CD69 expression in PBMCs from pigs that received a protein subunit vaccine. Following in vitro restimulation with the vaccine antigen, CD4 and CD8 T cells upregulated CD69, which makes CD69 not only a suitable marker for primed T cells but also for activation-induced marker (AIM) assays. Thus, Ankyron^™^ EG40360 expands the porcine immunology toolkit and will facilitate a more comprehensive characterization of activated T cells in vaccine development and infection studies.

## 1. Introduction

T cells are central players in protective immunity; yet the complexity and heterogeneity of T-cell populations in pigs remain underexplored. The identification of effector cells or cells with tissue-resident status are key elements to evaluate the development and activation of T cells and their contribution to protective immunity. In pigs, however, this identification has been challenging thus far due to a lack of commercially available monoclonal antibodies against porcine targets, particularly CD69.

In humans and mice, the type II C-lectin receptor CD69 serves two functions – as an early lymphocyte activation and a tissue-resident memory marker^1^. Since CD69 is rapidly expressed on the surface of T cells following a range of stimulations including mitogenic stimulations, TCR/CD3 engagement, and cytokines like type I interferons^2–5^, it has been extensively used in human and murine immunology to monitor activation kinetics and cell responsiveness^6^. Besides being upregulated in early activated cells, CD69 is also expressed on tissue resident memory T cells (TRMs). Consequently, TRMs are commonly identified by their expression of CD69, often in combination with CD103 and CD49a, two cell surface proteins that promote tissue retention^12^. TRMs have emerged as key mediators of robust immune responses at barrier sites and are increasingly being recognized as essential for controlling a range of pathogens including Influenza, Respiratory syncytial virus, *Chlamydia trachomatis*, *Helicobacter pylori* and *Mycobacterium tuberculosis* in mice and humans^7–11^. The critical role of CD69 in monitoring activation kinetics and the location and role of TRMs highlights the relevance of this receptor for biological research.

However, in pigs, the application of CD69 as a routine activation or tissue-residency marker has been relatively limited. In recent years, monoclonal antibodies specific to porcine CD69 have been developed by two research groups and successfully employed in the identification of lymphocyte activation and tissue-resident status of BAL CD8 T cells^13–16^. However, the availability of these antibodies through commercial suppliers remains limited, hampering research towards a better understanding of crucial immune cell processes. Moreover, the incorporation of anti-porcine antibodies into larger flow cytometry panels remains challenging due a limited availability of directly conjugated reagents, resulting in reliance on indirect labeling approaches with isotype constraints that increase complexity, cost and limit multiplexing. To help overcome these limitations, we evaluated a novel CD69 binding ankyrin repeat protein as a potential alternative tool for detecting and studying CD69 in pigs.

Ankyrin repeat proteins, one of the most common protein motifs in nature, are characterized by an array of 30–34 amino acid motifs that fold into a helix-turn-helix confirmation and form elongated structures^17,18^. These proteins can contain as many as 33 repeating motifs, though most commonly they include 2–6 repeats^19^. These repeats form a structure characterized by a hydrophobic core and a large, concave protein-binding surface^19^. The protein-binding surface with highly variable surface residues determines target recognition, allowing for specific, high-affinity interactions with a wide variety of target proteins^20^. This has been employed in the development of designed ankyrin repeat proteins (DARPins), where the non-conserved surface-exposed residues are randomized and used for the generation of synthetic combinatorial libraries. These libraries usually have a theoretical diversity of > 10^12^ unique sequences and can be subsequently screened for the high affinity binding of target proteins^21–23^. Ankyrin repeat proteins offer several practical advantages over monoclonal antibodies for staining applications such as flow cytometry and immunofluorescence microscopy. For example, owing to their much smaller size (~ 15 kDa compared with ~ 150 kDa for IgG), ankyrin repeat proteins enable improved tissue penetration. In addition, the absence of an Fc region may reduce nonspecific background, while the lack of isotypes simplifies integration into larger, high-parameter flow cytometry panels.

Using the above-described process, ProImmune Ltd. (Oxford, UK) generated the Ankyron^™^ clone EG40360 targeting porcine CD69. The binding was evaluated by target-specific immunoassays (ELISA), confirming a favorable signal-to-noise ratio of Ankyron^™^ EG40360 binding to immobilized porcine CD69 relative to the background signal from a non-cognate target protein. However, its suitability for application to porcine immune cells in flow cytometry and microscopy remained unvalidated. Here, we demonstrate that the commercially available Ankyron^™^ EG40360 specifically binds porcine CD69 and is suitable for both flow cytometry and microscopy, underscoring its strong potential to advance studies of porcine immune cell activation and differentiation into TRMs.

## 2. Material and Methods

### 2.1 Porcine CD69 transfected HEK293 T cells

For the confirmation of anti-CD69 Ankyron^™^ EG40360 binding to porcine CD69, a recombinant fusion protein of porcine CD69 with a C-terminal FLAG-tag was expressed in HEK293 T cells. To this end, a custom pcDNA3.1(+) expression vector containing the porcine CD69 coding sequence (NM_214091.2) with a C-terminal FLAG tag was obtained from BioCat (Heidelberg, Germany). Prior to transfection, HEK293T cells were propagated in DMEM supplemented with 1 mM sodium pyruvate, 100 U/mL penicillin, 0.1 mg/mL streptomycin (PAN Biotech, Aidenbach, Germany) and 10% fetal bovine serum (FBS, Merck KGaA, Darmstadt, Germany). Cells were then seeded into a 24-well cell culture plate. At a confluence of 70–80%, cells were transfected with K4 transfection system according to manufacturer’s instructions (Biontex Laboratories GmbH, Munich, Germany). In brief, 30 min before the addition of the lipoplex, 1% (v/v) K4 multiplier was added to the cells. During the incubation, 0.5 μg DNA was diluted in serum-free medium and combined with 2 μl K4^®^ Transfection Reagent in serum-free medium. After 20 min at room temperature (RT), the DNA-lipid complex was added to the cells and incubated for 24 h at 37°C with 5% CO2. Cells were then harvested with trypsin and analyzed by flow cytometry as described in 2.3. Non-treated HEK293T cells served as a negative control. For microscopy, HEK293T cells were seeded onto poly-D-lysin coated coverslips (Neuvitro Corporation, Camas, WA) before transfection. Microscopy staining was performed as described in 2.4.

### 2.2 Animals and lymphocyte isolation

Blood for ex vivo analyses was obtained from a nearby commercial slaughterhouse. In compliance with the Austrian Animal Welfare Slaughter Regulation, six-to-seven months old pigs were anesthetized by electrical stunning followed by exsanguination. Blood samples were collected directly into beakers containing heparin during routine slaughter procedures. As no experimental procedures were performed on live animals and no animals were sacrificed specifically for research purposes, ethical approval was not required. All procedures were conducted in accordance with relevant guidelines and regulations. After the isolation of peripheral blood mononuclear cells (PBMCs) by density gradient centrifugation using lymphocyte separation medium (Pancoll, PAN Biotech), the cells were counted on a Sysmex XP 300 hematology analyzer (Sysmex Europe GmbH, Norderstedt, Germany) and frozen in freezing media (50% RPMI 1640 (PAN Biotech), 40% FBS (Merck KGaA), 10% DMSO (Merck KGaA)) at − 150°C for future use.

For the flow cytometry assay measuring changes in CD69 expression upon vaccine-antigen restimulation, cells from vaccinated or unvaccinated pigs were utilized. The vaccination trial was conducted by the previous research group of the last author at the Department of Population Health and Pathobiology, College of Veterinary Medicine at North Carolina State University and approved by the North Carolina State University Institutional Animal Care and Use Committee (IACUC; ID# 21–199B; approval date: 13 May 2021). All experiments were performed in accordance with relevant guidelines and regulations and details of the study can be obtained from Proctor et al., 2024^24^. In brief, 7-week-old pigs received two intramuscular (IM) and two intranasal (IN) vaccinations at 7-day intervals with the subunit vaccine composed of TriAdj-adjuvanted CPAF protein (Chlamydial Protease Activity Factor). Per dose, pigs received the TriAdj adjuvant consisting of 150 μg of poly I:C, 300 μg of a host defence peptide, and 150 μg of polyphosphazene (provided by the Vaccine and Infectious Disease Organization, Saskatoon, SK, Canada) formulated with 30 μg of CPAF. MOCK pigs received the TriAdj adjuvant without CPAF. Throughout the study, blood was collected into heparin vacutainers (BD Bioscience, San Jose, CA, USA) and PBMC isolation was carried out using Sepmate tubes (StemCell, Vancouver, BC, Canada) and Ficoll-Paque (GE Healthcare, Uppsala, Sweden). PBMCs were then cryopreserved in freezing media, stored in liquid nitrogen, transported on dry ice to the University of Veterinary Medicine Vienna, and kept there at −150°C until antigen restimulation was carried out as outlined in 2.3. At the end of the trial period, animals were sedated with TKX (Telazol, ketamine, and xylazine; 0.04 mL/kg IM) prior to euthanasia by intravenous administration of Euthasol (390 mg/mL pentobarbital and 50 mg/mL phenytoin; 1 mL/10 lbs IV).

Before using the PBMCs for downstream methods, the cells were thawed, washed twice, and resuspended in cell culture medium (RPMI 1640; PAN Biotech) supplemented with 10% FBS (Merck KGaA) and Penicillin/Streptomycin (PAN Biotech).

### 2.3 Cell stimulation and Flow Cytometry

For the cell surface staining of CD69 on transfected HEK293T cells (section 2.1), cells were harvested by trypsinization 24 h after transfection. For the cell surface staining of CD69 on PBMCs, round-bottom 96-well plates were seeded in quadruplicates with 5 × 10^5^ thawed PBMCs per well in culture medium (RPMI 1640 + 10% FBS + Penicillin/Streptomycin). PBMCs were either left unstimulated (negative control) or stimulated overnight with PMA (5 ng/ml) and ionomycin (500 ng/ml) at 37°C and 5% CO2. PBMCs from the vaccination trial were stimulated using media (negative control) or an overlapping 15-mer CPAF peptide pool (1 μg/mL per peptide; PepMix^™^ CPAF, JPT Peptide Technologies, Berlin, Germany). Cells stimulated with ConA (3 μg/mL) served as positive controls. After 16 h of culture, Brefeldin A (BD GolgiPlug^™^, BD Biosciences) was added at a final concentration of 1 μg/mL for 4h.

After 20 h of culture, quadruplicate wells were pooled and stained for flow cytometry as described below. Before using the V5-tagged Ankyron^™^ EG40360, it was pre-conjugated to an anti-V5 reagent according to manufactures instructions (ProImmune Ltd). Briefly, per sample, 0.5 μg Ankyron^™^ EG40360 was mixed with 6 μl of the anti-V5 reagent in a total volume of 12 μl and incubated for 30 min at 4°C. PBMCs or HEK293T cells were first stained with primary monoclonal antibodies, including directly conjugated antibodies and the pre-conjugated Ankyron^™^ EG40360 (0.5 μg/sample) in staining buffer (PBS + 2% FBS). After two washes, this was followed by staining with secondary antibodies and the fixable Viability Dye eFluor^™^ 780 or 506 (Thermo Fisher Scientific, Waltham, MA, USA) to exclude dead cells. All staining steps were carried out at 4°C for 30 min. Antibody and Ankyron^™^ details are listed in Table 1. For the panel “Antigen specific CD69 expression”, which required intracellular cytokine staining, cells were fixed and permeabilized using the BD Cytofix/Cytoperm^™^ Fixation/Permeabilization Kit (BD Biosciences) according to the manufacturer’s instructions. The intracellular staining step was carried out for 30 min at 4°C.

Antibodies and Ankyron^™^ were titrated prior to use, and compensation was calculated from single-color controls. A minimum of 2 × 10^5^ events per sample were acquired for mitogen stimulation experiments and 5 × 10^5^ events for antigen-specific assays using a Beckman Coulter CytoFLEX LX^™^ (U3-V5-B3-Y5-R3-I2 laser configuration). Data were analyzed in FlowJo^™^ Software (v10.10.0; BD Biosciences) using gates defined by fluorescence minus one (FMO) controls. Dead cells and doublets were excluded as shown in **Supplementary Fig. 1–5**.

### 2.4 Fluorescence Microscopy

Porcine PBMCs were stimulated for 18 h with PMA/ionomycin at 5 ng/ml and 500 ng/ml respectively or left unstimulated. PBMCs were adhered to glass coverslips by gravity sedimentation shortly before the staining as described by Tsang et al. (2017)^25^. Briefly, cells were harvested and resuspended in PBS at 1 × 10^6^ cells/ml with 500 μl added to each well of a 24-well plate containing a coverslip. Cells were allowed to settle and adhere for 30 min at RT. After removal of nonadherent cells, remaining cells were fixed with 1% formaldehyde for 10 min at RT, followed by one wash with PBS. HEK293T cells (2.1) were seeded onto poly-D-lysin coated coverslips (Neuvitro Corporation) before transfection and transfection was carried out as described in 2.1 After an incubation for 24 h, cell culture media was removed, and cells were washed with PBS and fixed as described above. After fixation, cells (PBMCs or HEK293T cells) were blocked with PBS + 2% FBS for 30 min and incubated for 45 min at RT with 200ul of the pre-conjugated anti-CD69 Ankyron^™^ EG40360 (1 μg/well; APC) and, in case of HEK293T cells, with an anti-FLAG-FITC antibody (clone M2, Merck KGaA). Pre-conjugation of the Ankyron^™^ EG40360 was performed as described under 2.3. After two washes with PBS, nuclei were counterstained with DAPI (Thermo Fisher Scientific) for 15 min, followed by two more washes with PBS. Coverslips were then mounted onto microscope slides with ProLong Diamond antifade mounting media (Thermo Fisher Scientific). Images were acquired with an upright fluorescence microscope (ZEISS Axio Imager Z2) using a 40× oil-immersion objective with or without a 1.6× lens as indicated in the figure legends. The following filters were used to detect DAPI and the fluorochromes FITC and APC: #49 Ex 365 | Em 445/50, #38 Ex 470/40 | Em 525/50, #50 Ex 640/30 | Em 690/50. Image acquisition and analysis were performed using ZEISS ZEN software (version 3.10.103.01).

### 2.5 PrimeFlow^™^ RNA Assay

The PrimeFlow^™^ RNA Assay enables the simultaneous detection of RNA and proteins targets and was used for the detection of CD69 mRNA and CD69 protein on live PBMCs and T cells. For the analysis of CD69 mRNA and protein kinetics, PBMCs were thawed and stimulated with PMA (5 ng/ml) and ionomycin (500 ng/ml). After 1, 2, 4, 8 and 24 h cells were harvested and stained as described under 2.2 and outlined in Table 1. Following this cell surface staining, the PrimeFlow^™^ assay was performed following manufacturers instructions (Thermo Fisher Scientific). Briefly, cells were fixed for 30 min at 4°C with Fixation Buffer 1, washed twice in permeabilization buffer, followed by a 60 min incubation at RT in Fixation Buffer 2. After two more washing steps, cells were incubated with the porcine CD69 mRNA specific target probe for 2 h at 40°C in a hybridization chamber. The target probe was designed by Thermo Fisher based on the CD69 mRNA sequence NM_214091.2 (Assay ID: VF1–4294934-PF, Thermo Fisher Scientific). Following three washing steps, cells were resuspended in PrimeFlow^™^ RNA Assay wash buffer with RNase inhibitors and stored overnight at 4°C. The next day, two amplification steps (1.5 h at 40°C) with in-between washes were performed before the addition of the Alexa Fluor^™^ 647-conjugated label probe for 1 h at 40°C. After three final washes, cells were resuspended in staining buffer (PBS + 2% FBS) and acquired on a Beckman Coulter CytoFLEX LX^™^ (U3-V5-B3-Y5-R3-I2 laser configuration).

### 2.6 Statistical analysis

Statistical analyses were performed using GraphPad Prism 11.0.0 (Graph Pad Software, San Diego, CA, USA). Responses to in vitro stimulation (media vs. antigen) across MOCK and VACC groups were analyzed using a two-way repeated-measures (RM) ANOVA. Pairwise comparisons were conducted using Bonferroni multiple comparison test. Statistical significance was defined as p ≤ 0.05 (✳), p ≤ 0.01 (✳✳), and p ≤ 0.001 (✳✳✳).

## 3. Results and Discussion

### 3.1 CD69 Ankyron^™^ EG40360 binding specificity on transfected HEK293T cells

**Figure 1:**
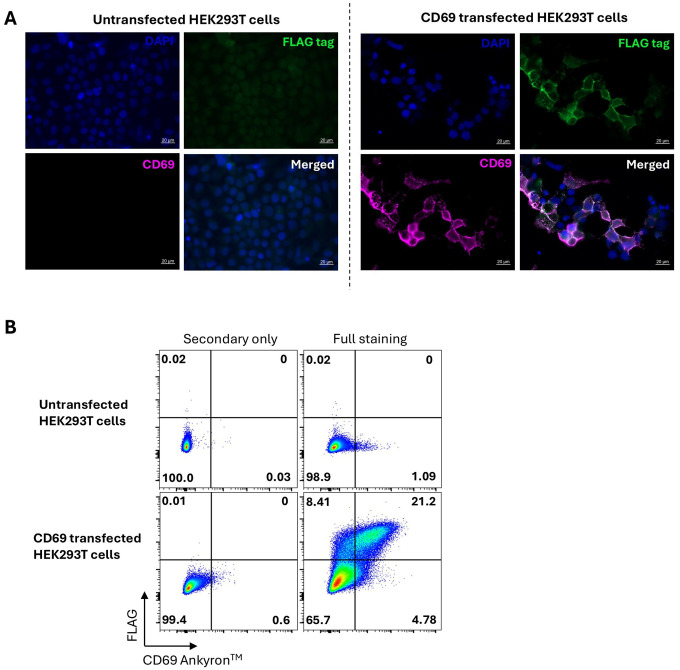
Porcine CD69 Ankyron^™^ binding specificity on transfected HEK293T cells. HEK293T cells were transfected with an expression vector coding for porcine CD69 including a C-terminal FLAG tag. Non-transfected cells were used as a control. After 24h, cells were stained using CD69 Ankyron^™^ (V5 tagged) with anti-V5 APC and anti-FLAG specific mAbs for immunofluorescence microscopy (**A**) or flow cytometry (**B**). Fluorescence microscopy images were acquired with an upright fluorescence microscope (Axio Imager Z2) using a 40× oil-immersion objective.

To verify binding of CD69 Ankyron^™^ EG40360 to porcine CD69, HEK293T cells were transiently transfected to express recombinant porcine CD69 with a C-terminal FLAG tag. Immunofluorescence microscopy and flow cytometry were used to analyze transfected and untransfected HEK293T cells stained with anti-porcine CD69 Ankyron^™^ and anti-FLAG monoclonal antibodies (mAbs) (Fig. 1). As expected, untransfected HEK293T cells showed minimal to no staining with either the CD69 Ankyron^™^ or anti-FLAG mAbs by microscopy or flow cytometry (Fig. 1A,B). In contrast, transfected cells showed clear co-staining of the CD69 Ankyron^™^ and the anti-FLAG mAbs (Fig. 1A,B). Flow cytometric analysis demonstrated that 21.2% of transfected cells were double-positive, indicating correct expression and folding of recombinant porcine CD69 and confirming specific binding of CD69 Ankyron^™^ to porcine CD69 (Fig. 1B).

### 3.2 PMA/ionomycin induced activation of circulating T cells can be detected using the porcine CD69 Ankyron^™^

**Figure 2:**
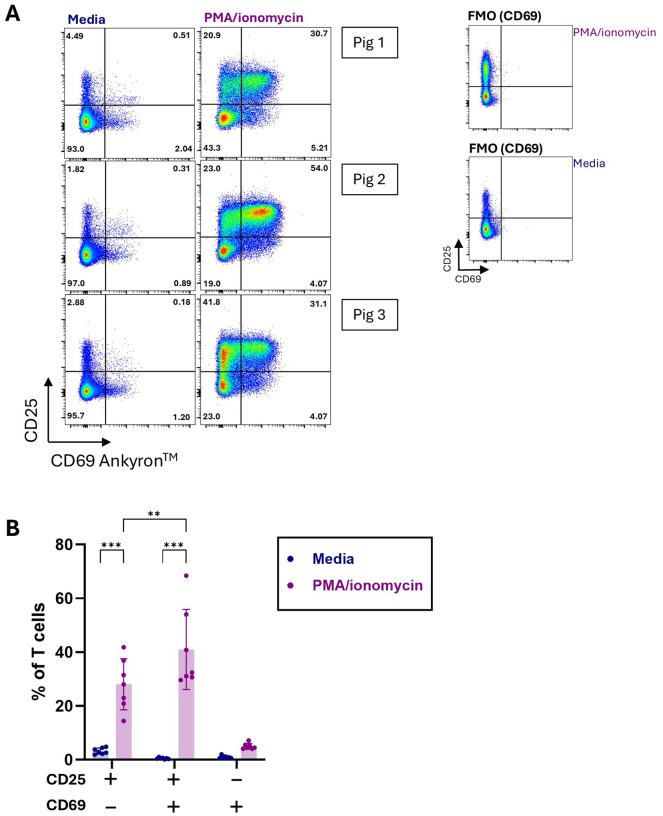
Expression of CD69 on circulating T cells after stimulation with PMA/ionomycin, as determined by flow cytometry. Cryopreserved PBMCs were thawed and stimulated with PMA/ionomycin for 18h or left unstimulated (media). Surface staining was performed with antibodies against CD3 and CD25 and porcine CD69 Ankyron^™^. During data analysis, dead cells and doublets were excluded, as shown in **Supplementary Figure 2**. After identification of T cells (CD3+), their expression of CD25 and CD69 was analyzed as shown for three representative pigs in **(A)**. The plots on the right show the CD69 FMO control for media and PMA/ionomycin stimulated cells. **(B)** shows the percentage of each subset (CD25+CD69-, CD25+CD69+, CD25-CD69-) after PMA/ionomycin stimulation or left unstimulated for 18 h. Each symbol represents data from one individual pig (n=7). The statistical analysis was performed via GraphPad Prism using 2-way RM ANOVA and Bonferroni multiple comparisons test. *** p < 0.001.

Following confirmation of CD69 Ankyron^™^ binding to CD69-transfected cells, its staining of porcine lymphocytes isolated from peripheral blood was evaluated by flow cytometry. Given recent findings demonstrating strong upregulation of CD69 on porcine T cells following PMA and ionomycin stimulation that is consistent with observations in human T cells, we assessed the performance of CD69 Ankyron^™^ under comparable activation conditions^15^. PBMCs were cultured overnight in the presence of a low concentration of PMA [5 ng/ml] and ionomycin [500 ng/ml] or left unstimulated. While a low (co-) expression of CD69 and CD25 was observed on unstimulated T cells, PMA/ionomycin stimulation strongly upregulated the (co-)expression of CD25 and CD69 as detected by the ankyrin repeat protein (Fig. 2A). The predominant phenotype induced by PMA/ionomycin was CD25^+^CD69^+^ (mean of 39.7%), followed by cells expressing CD25 but no CD69 (mean of 31.4%) (Fig. 2B). After the 18 hours of stimulation, only a small fraction of T cells expressed CD69 without co-expression of CD25, and this proportion was comparable to that observed in unstimulated cells. The marked CD69 upregulation observed under polyclonal PMA/ionomycin stimulation and detected by CD69 Ankyron^™^ binding is consistent with previously reported results obtained using CD69 mAbs^13,15,16^.

**Figure 3:**
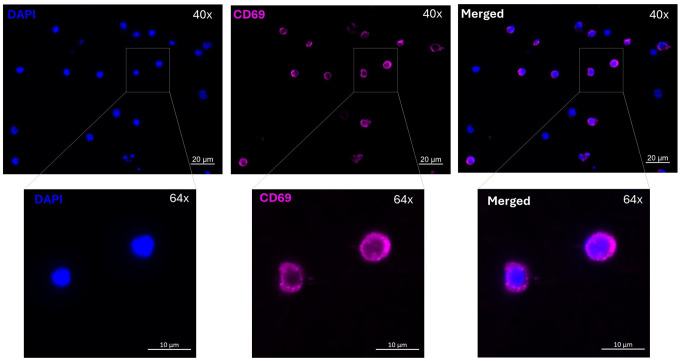
Expression of CD69 on porcine PBMCs visualized by fluorescence microscopy. Cryopreserved PBMCs were thawed and stimulated with PMA/ionomycin for 18h. After which cells were harvested and adhered to a coverslip. Immunofluorescent staining was performed with porcine anti-CD69 Ankyron^™^ tagged with V5 and pre-conjugated to anti-V5 APC (pink). Cell nuclei were counterstained with DAPI (blue). Images were acquired with an upright fluorescence microscope (Axio Imager Z2) using a 40× oil-immersion objective. Images shown in the bottom panel were acquired usinga 40× objective with a 1.6× lens (total 64×).

Tian et al. (2022) reported about 40% CD69^+^ cells within CD8α T cells and about 55% CD69^+^ cells within CD4 T cells following a 6h stimulation with PMA [50 ng/ml] and ionomycin [500 ng/ml], while Moorton et al. (2024) indicated between 74–94% CD69^+^ T cells after 18h culture with a lower concentration of PMA/ionomycin^15,16^. Despite some pig or culture protocol specific variability in the extent of CD69 upregulation following polyclonal stimulation, a significant upregulation was consistently reported by all studies. We further visualized the extracellular binding of CD69 Ankyron^™^ to porcine PBMCs by fluorescence microscopy thereby confirming its suitability for fluorescence-based imaging applications and supporting its use in future microscopy studies (Fig. 3). Overall, the staining pattern observed with the CD69 Ankyron^™^ was consistent with previously reported patterns for CD69 mAbs, where polyclonally stimulated T cells frequently co-express CD69 and CD25^15^.

Cryopreserved PBMCs were thawed and stimulated with PMA/ionomycin for 18h. After which cells were harvested and adhered to a coverslip. Immunofluorescent staining was performed with porcine anti-CD69 Ankyron^™^ tagged with V5 and pre-conjugated to anti-V5 APC (pink). Cell nuclei were counterstained with DAPI (blue). Images were acquired with an upright fluorescence microscope (Axio Imager Z2) using a 40× oil-immersion objective. Images shown in the bottom panel were acquired using a 40× objective with a 1.6× lens (total 64×).

### 3.3 CD69 expression precedes CD25 making CD69 an early activation marker in pigs

CD69 is a well-established early activation marker of human lymphocytes, with upregulation detectable as early as 4 h following anti-CD3/CD28 stimulation or even within 2 h after high-dose of PMA/ionomycin^26–28^. However, due to the limited availability of species-specific reagents, the kinetics of CD69 induction in porcine lymphocytes remain poorly characterized, and its suitability as an early activation marker in this species remains unclear. Hence, we set out to characterize the kinetics of CD69 mRNA and protein expression following polyclonal stimulation, using the Prime Flow assay for simultaneous detection of CD69 mRNA and CD69 protein (stained with CD69 Ankyron^™^).

**Figure 4:**
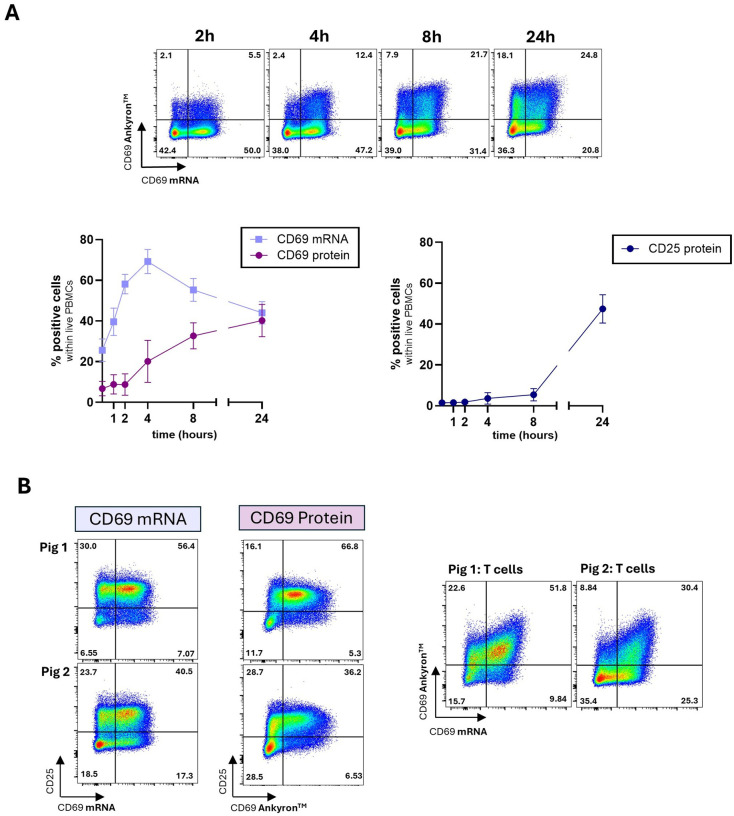
Expression kinetics of CD69 mRNA, and CD69 and CD25 protein upon mitogen stimulation. Cryopreserved PBMCs were thawed and stimulated with PMA/ionomycin for 0–24 hours **(A)** or 18h **(B)**. Surface staining was performed with antibodies against CD3, CD25 and porcine CD69 Ankyron^™^, followed by the detection of CD69 mRNA with the PrimeFlow^™^ RNA assay. The graphs depicted in **(A)** show the kinetics of CD69 transcription and translation and the CD25 expression within live PBMCs. The data in the graphs represent the mean ± SD of 4 pigs. Representative flow cytometry plots of CD69 protein (Ankyron^™^) and CD69 mRNA expression within live cells are shown. **(B)** shows representative flow cytometry plots of gated T cells and their CD25/CD69 mRNA and CD25/CD69 protein expression upon 18h PMA/ionomycin stimulation. The flow cytometry plots on the right show the expression of CD69 mRNA and protein within T cells for two representative pigs. The gating strategy is shown in **Supplementary Figure 3** and **4**.

At the start of the culture, 21% of PBMC expressed CD69 mRNA. Upon simulation with PMA/ionomycin, this frequency of CD69 mRNA-positive PBMCs quickly and substantially increased within an hour to 30–40%; it peaked at 4 h post-stimulation with approximately 66%, followed by a gradual decline (Fig. 4A).

This timeline is consistent with a previous study examining CD69 mRNA expression in porcine leukocytes following PMA stimulation, which reported maximal expression at 6 h and a declined but sustained expression through the end of the culture period (24 h)^29^. Regarding the co-expression of CD69 mRNA and protein, most cells expressed CD69 mRNA in the absence of CD69 protein at 2 h poststimulation, as shown in the pseudocolor plot (Fig. 4A). CD69 protein expression increased progressively from 4 h onward. Consequently, cells started to co-express CD69 mRNA and extracellular CD69 protein at 4 h (upper right quadrant of the pseudocolor plot). At the latest timepoint (24 h), an increasing proportion of cells were positive for CD69 protein but negative for CD69 mRNA (upper left quadrant pseudocolor plot; Fig. 4A). The discrepancy between declining CD69 mRNA levels and increasing surface protein expression is most likely attributable to the prolonged persistence of CD69 protein on the cell surface following activation while mRNA is only transiently expressed^30,31^.

While CD69 is known to be expressed very early following lymphocyte activation, CD25 is classified as a mid- to late-stage activation marker in humans and mice^32^. To validate this classification in porcine cells, we included CD25 in our time-course analysis and found that no substantial CD25 protein expression was detectable within the first 8 h after stimulation (Fig. 4A). While about 40% of lymphocytes were positive for CD69 protein at 8 h, CD25 expression remained at low baseline levels (< 3%). By 24 h, however, CD25 expression increased strongly, closely matching the frequency of CD69^+^ cells (Fig. 4A). Similar patterns have been observed for human CD4^+^ and CD8^+^ T cells upon CD3/28/IL-2 stimulation, where CD25 expression only increased at later timepoints (> 8 h)^28^. After the confirmation of these expression kinetics in live PBMCs, we evaluated the co-expression of CD25 with CD69 mRNA and protein within live porcine T cells at 18 h. At this time point, most T cells co-expressed CD69 mRNA and CD69 protein, although individual variation was observed in the frequency of co-stained cells (Fig. 4B). This analysis also confirmed that at later time points (18 h), nearly all T cells expressing CD69 mRNA/CD69 protein co-express CD25 (Fig. 2, Fig. 4B). Overall, these data confirm CD69 as an early activation marker in pigs and support the classification of CD25 as a later-expressed marker.

### 3.4 Antigen-specific restimulation induces expression of CD69 and CD25

**Figure 5:**
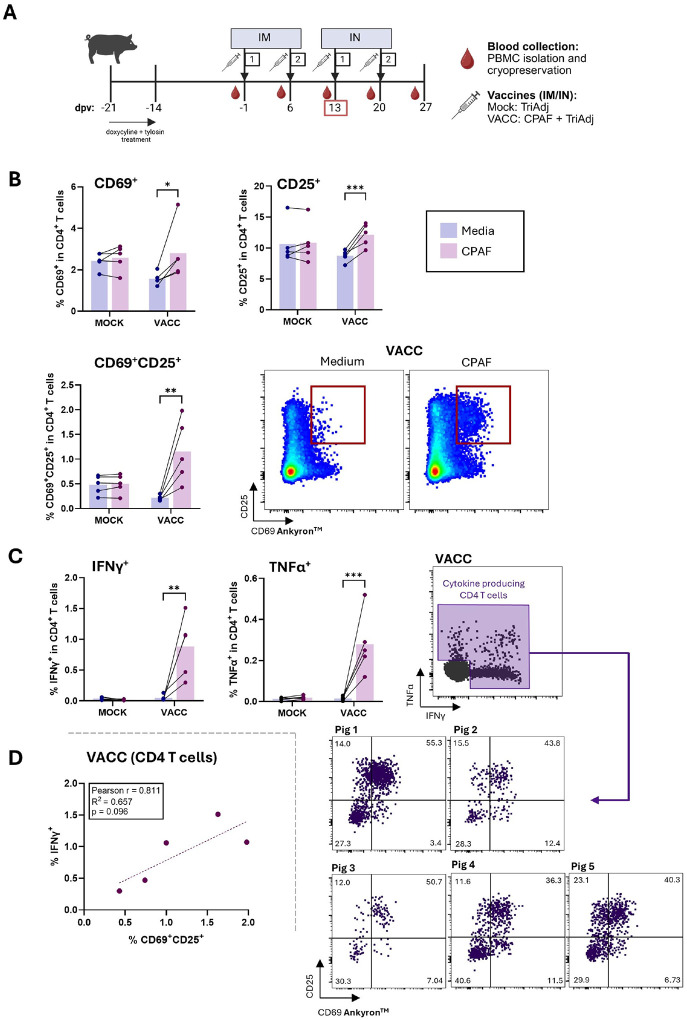
In vitro stimulation with vaccine antigen (CPAF) leads to upregulation of CD69/CD25 on CD4 T cellsfrom vaccinated pigs. Pigs were either vaccinated with CPAF adjuvanted with TriAdj (VACC) or received TriAdj adjuvant alone (MOCK) as outlined in **(A)**. Cryopreserved PBMCs from day 13 post-vaccination were thawed and stimulated with an overlapping CPAF peptide pool or left unstimulated (media control)for 20 hours. Following gating on live and singlet cells, CD4 T cells were identified and analyzed for activation marker and cytokine expression as shown in **Supplementary Figure 5**. **(B)** shows the expression of CD69 and CD25 on CD4 T cells from MOCK and VACC groups, with or without CPAF stimulation. Representative flow plots depict the CD25 and CD69 expression by CD4 T cells from one vaccinated pig. The red box highlights CD25^+^CD69^+^ double-positive CD4 T cells. **(C)** Frequencies of IFNγ^+^ or TNFα^+^ CD4 T cells in unstimulated and CPAF-stimulated samples. Cytokine producing CD4 T cells from vaccinated pigs were further analyzed for their CD25/CD69 phenotype, as shown in the flow plots. **(D)** shows the correlation between the frequency of IFNγ^+^ cells and CD69^+^CD25^+^ cells within CD4 T cells from vaccinated pigs. Pearson’s correlation test was used to assess the correlation with Pearson r, R^2^ and p-value indicated in the graph. For visual representation, the dashed line was added to indicate the linear trend. Each dot in the bar graphs represents one individual pig (n = 5 per group). The statistical analysis (B-C) was performed using GraphPad Prism, applying two-way RM ANOVA with Bonferroni multiple comparisons test. The statistical analysis of within-group comparisons is shown. * p < 0.05, ** p < 0.01, *** p < 0.001. dpv = days post (first) vaccination. Vaccination trial timeline in (A) was created in BioRender. Kaeser, T. (2026) https://BioRender.com/yz8rtue.

Following confirmation through transfection and PMA/ionomycin stimulation experiments that the CD69 Ankyron^™^ effectively binds porcine CD69 and that CD69 serves as an early activation marker in pigs, we next sought to determine whether CD69 could be used to detect antigen-specific T cells postvaccination. To that end, we utilized samples from a previously published vaccination trial^24^. In brief, pigs were either vaccinated with TriAdj-adjuvanted CPAF (VACC) or received the TriAdj adjuvant without CPAF (MOCK) as outlined in Fig. 5A. PBMCs from 13 days post (first) vaccination were thawed and either left unstimulated (media control), or stimulated overnight (20 h) with a CPAF peptide pool. Then, T cells were analyzed for their expression of activation induced markers (CD69, CD25) and cytokine production (IFNγ, TNFα).

While CD69 expression in MOCK pigs remained unchanged irrespective of in vitro CPAF stimulation, T cells from vaccinated pigs showed a slight but significant increase in the frequency of CD69^+^ CD4 T cells upon CPAF restimulation (Fig. 5B). A similar pattern was observed for CD25 expression on CD4 T cells with higher expression on cells from vaccinated pigs following antigen restimulation (Fig. 5B). This suggests that both CD25 and CD69 are upregulated on antigen-specific T cells upon restimulation and may therefore serve as useful markers of antigen-specific activation. Hence, we analyzed the co-expression of CD69 and CD25 on CD4 T cells: while media controls and MOCK animals maintained relatively low background levels of CD69^+^CD25^+^ CD4 T cells, this combined phenotype showed a pronounced increase in vaccinated pigs following CPAF restimulation (Fig. 5B). A similar increase was identified in CD8 T cells, where CD69 and CD25 co-expression seemed to be associated with a clearer distinction of antigen-specific responses compared to either marker alone (**Supplementary Fig. 6**). In line with this observation, Moorton and colleagues identified the CD25^+^CD69^+^ phenotype as one of the most prominent activation-induced markers (AIMs) expanded in response to overnight ASFV restimulation in PBMC samples from ASFV-challenged pigs^15^.

The increased expression of the AIMs CD69 and CD25 in the VACC group closely paralleled the production of IFNγ and TNFα by these animals (Fig. 5C). To further explore the relationship between these two parameters, the CD25/CD69 phenotype of cytokine-producing CD4 T cells was analyzed (Fig. 5C). While some variation was observed between individual pigs, cytokine-producing CD4 T cells were generally consistent in falling into two main phenotypes: CD69^+^CD25^+^ (36.3–55.3%) or CD69^−^CD25(27.3–40.6%). Conversely, within the CD69^+^CD25^+^ CD4 T-cell population, a mean of 46.7% were positive for IFNγ production (**Supplementary Fig. 7**). This is consistent with previous studies in human T cells, where AIM and intracellular cytokine assays do not show perfect overlap and are preferably used in combination to better capture the breadth and functional diversity of antigen-specific T cells responses^33–35^. Unlike cytokine assays, AIM assays are not restricted to the detection of specific cytokines and can capture a broader spectrum of antigen-specific T cells, including non-cytokine producers, often resulting in improved specificity^34,36^. The absence of AIM marker expression (CD69/CD25) on some of the cytokine-producing CD4 T cells in our study (Fig. 5C) may result from the addition of Brefeldin A during the final 4 h of the 20 h culture. Brefeldin A not only inhibits cytokine secretion but can also prevent the surface expression of AIMs, like CD69, when present throughout the stimulation period^37^. However, our initial tests comparing the presence or absence of protein transport inhibitors showed no impact on CD69 or CD25 expression when the inhibitors were added during the final 4 h of an overnight antigen stimulation (data not shown). Similarly, Wan et al. reported that surface expression of CD25/CD69 on human CD4 T cells remained largely unchanged regardless of inhibitor treatment, using a culture protocol similar to ours^35^. Another explanation for the partial absence of the AIM phenotype among cytokine-producing CD4 T cells could be that the CD69/CD25 combination does not fully capture all activation-induced phenotypes in pigs. Additional AIM markers, such as CD40L, previously used in pigs by Moorton and colleagues, or OX40 and 4–1BB, which are established in human T-cell assays, may complement this panel and enable a more comprehensive detection of antigeninduced activation^15,38^.

Notably, vaccinated pigs exhibiting a low to moderate increase of the CD69^+^CD25^+^ phenotype also produced IFNγ at correspondingly low to moderate levels, while the high responders showed the most pronounced upregulation of CD69 and CD25 expression alongside the highest levels of IFNγ production (Fig. 5D). This highlights that both AIM expression and cytokine production can be utilized to identify antigen-specific T cells.

Overall, our results confirm that the combination of CD69 and CD25 can be used to identify antigen-specific T cells (CD4 and CD8) and that the CD69 Ankyron^™^ can be effectively used as a detection reagent in this antigen-specific context.

## 4. Conclusion

In this study, we demonstrate that Ankyron^™^ EG40360 (ProImmune Ltd.) specifically binds porcine CD69 and can be utilized in assays involving flow cytometry and microscopy. We confirmed CD69 as an early marker of lymphocyte activation in pigs, detectable as early as 4 h post-stimulation. In contrast, CD25 can be classified as a mid- to late-stage activation marker. Using the Ankyron^™^ EG403060 we further identified the co-expression of CD69 and CD25 as a robust AIM combination for the reliable detection of antigen-specific T cells post vaccination. This will enable us now to integrate CD69-based AIM assays with conventional intracellular cytokine approaches to assess antigen-specific T-cell responses in pigs. One key advantage of this approach is that, as a surface marker, CD69 can be detected without fixation or permeabilization, allowing the T cells identified via CD69-based assays to be used in downstream applications such as sorting, functional test or transcriptomic analysis. Overall, the validation of the CD69 Ankyron^™^ EG403060 as a detection reagent significantly enhances the porcine immunology toolkit and will enable a more comprehensive identification of activated T cells in vaccine development and infection studies.

## Supplementary Material

Supplementary Files

This is a list of supplementary files associated with this preprint. Click to download.

- Bettinetal.2026CD69SupplementaryMaterial.pdf

## Figures and Tables

**Table 1:** Primary antibodies and secondary reagents used for flow cytometric analysis.

Antigen	Clone	Isotype	Fluoro-chrome	Labeling Strategy	Primary reagent source	Secondary reagent source
*CD69 transfected HEK293 T cells*						
CD69	EG40360	-	APC	Pre-conjugated	Prolmmune	Prolmmune
FLAG	M2	mlgG1	BV421	Biotin-Streptavidin	Sigma-Aldrich	BioLegend
Live/Dead	-	-	eFlour780	-	Invitrogen	-
*CD69 Ankyron^™^ EG40360 staining of porcine T cells (PBMCs)*						
CD3	PPT3	mlgG1	FITC	Directly conjugated	Southern Biotech	-
CD25	K231.3B2	mlgG1	BUV395	Biotin-Streptavidin	In house	BD Biosciences
CD69	EG40360	-	APC or PE	Pre-conjugated	Prolmmune	Prolmmune
Live/Dead	-	-	eFlour780	-	Invitrogen	-
*CD69 Ankyron^™^ EG40360 and CD69 mRNA (PrimeFlow^™^ assay)*						
CD3	PPT3	mlgG1	FlTC	Directly conjugated	Southern Biotech	-
CD25	K231.3B2	mlgG1	BUV395	Biotin-Streptavidin	In house	BD Biosciences
CD69	EG40360	-	PE	Pre-conjugated	Prolmmune	Prolmmune
CD69 mRNA	-	-	AF647	-	Thermo Fisher	Thermo Fisher
Live/Dead	-	-	eFlour780	-	Invitrogen	-
*Antigen-specific expression of CD69*						
CD3	PPT3	mlgG1	FITC	Directly conjugated	Southern Biotech	-
CD4	74-12-4	mlgG2b	PerCPCy5.5	Directly conjugated	BD Biosciences	-
CD8a	76-2-11	mlgG2a	BV421	Indirect	In house	BD Biosciences
δTCR	PPT16	mlgG2b	PECy7	Biotin-Streptavidin	In house	Thermo Fisher
CD25	K231.3B2	mlgGI	SBUV400	Directly conjugated	BD Biosciences	-
CD69	EG40360	-	APC	Pre-conjugated	Prolmmune	Prolmmune
Live/Dead	-	-	eFlour506	-	Invitrogen	-
IFNγ	P2G10	mlgGI	PE	Directly conjugated	BD Biosciences	-
TNFα	MAb11	mlgGI	BV605	Directly conjugated	BD Biosciences	-

## Data Availability

All data generated or analysed during this study are included in this published article (and its Supplementary files). Raw datasets are available from the corresponding author on reasonable request.
